# Supplementary material for: Reconciling Mining with the Conservation of Cave Biodiversity: A Quantitative Baseline to Help Establish Conservation Priorities
Source: PLoS One. 2016 Dec 20;11(12):e0168348. doi: 10.1371/journal.pone.0168348 (PMC5173368; doi:10.1371/journal.pone.0168348)
Supplement: S1 Dataset — (ZIP) [file pone.0168348.s002.zip › Taxa/Serra Norte/SN_2013/N4WS/Lista N4WS-59.pdf]

CAVIDADE N4WS-0059

Classe	Ordem	Fam/Outros	Gên/Outros	Espécie	S-U	U-S	Zon.
Arachnida	Acari	Ixodidae	*Amblyomma*	sp.1		X	P
Arachnida	Amblypygi	Phrynidae	*Heterophrynus*	*longicornis*		1	E
Arachnida	Araneae	Araneidae	*Alpaida*	*septemmammata*	X	X	E
Arachnida	Araneae	Corinnidae		jovem	1		E
Arachnida	Araneae	Ochyroceratidae	*Ochyrocera*	sp.1	X		E
Arachnida	Araneae	Pholcidae	*Mesabolivar*	*eberhard*	X	X	E
Arachnida	Araneae	Scytodidae	*Scytodes*	jovem	1		E
Arachnida	Araneae	Theridiidae	*Achaearanea*	sp.1		X	E
Arachnida	Araneae	Theridiidae	*Theridion*	sp.1	X		E
Arachnida	Araneae	Theridiosomatidae		jovem	X		E
Entognatha	Collembola	Entomobryidae		sp.6		X	E
Insecta	Blattodea			jovem		1	E
Insecta	Coleoptera	Carabidae		sp.5	X		E
Insecta	Diptera	Cecidomyiidae	Cecidomyiinae	sp.	X		E
Insecta	Diptera	Chloropidae		sp.		X	E
Insecta	Diptera	Culicidae	*Culex*	sp.	X	X	E/P
Insecta	Diptera	Keroplatidae	*Euceroplatus*	sp.		X	P
Insecta	Diptera	Limoniidae		sp.	X		E
Insecta	Diptera	Phoridae	Metopininae	sp.	X		E
Insecta	Diptera	Sciaridae	*Bradysia*	sp.	X		E
Insecta	Diptera	Tipulidae	Tipulinae	sp.	X		E
Insecta	Heteroptera	Reduviidae	Emesinae	jovem		1	E
Insecta	Hymenoptera	Formicidae	*Pachycondyla*	*striata*		2	E
Insecta	Hymenoptera	Formicidae	*Solenopsis*	sp.2	X		E
Insecta	Hymenoptera	Pemphredonidae		sp.1		X	P
Insecta	Hymenoptera	Vespidae		sp.1		1	E
Insecta	Isoptera	Termitidae	*Microcerotermes*	sp.	X	X	E
Insecta	Lepidoptera	Noctuoidea		sp.1		2	E
Insecta	Lepidoptera	Noctuoidea		sp.2	X	X	E/P
Insecta	Lepidoptera	Tineoidea		sp.3		X	E
Insecta	Neuroptera	Myrmeleontidae		jovem	2		E
Insecta	Orthoptera	Phalangopsidae	*Phalangopsis*	sp.		1	E
Insecta	Psocoptera	Ptiloneuridae	*Triplocania*	sp.1	X	X	E
Malacostraca	Isopoda	Dubioniscidae		sp.1	X		E
Amphibia	Anura	Strabomantidae	*Pristimantis*	cf. *fenestratus*	1		E
Amphibia	Anura			sp.2	1		E
Mammalia	Chiroptera	Emballonuridae	*Peropteryx*	sp.	3	3	E/P
Mammalia	Rodentia	Cricetidae	*Rhipidomys*	sp.	1		E
